# The haptic and the visual flash-lag effect and the role of flash characteristics

**DOI:** 10.1371/journal.pone.0189291

**Published:** 2018-01-03

**Authors:** Knut Drewing, Elena Hitzel, Lisa Scocchia

**Affiliations:** 1 Department of General Psychology, Justus-Liebig-University, Giessen, Germany; 2 Department of Psychology, University of Milano-Bicocca, Milano, Italy; Centre de neuroscience cognitive, FRANCE

## Abstract

When a short flash occurs in spatial alignment with a moving object, the moving object is seen ahead the stationary one. Similar to this visual “flash-lag effect” (FLE) it has been recently observed for the haptic sense that participants judge a moving hand to be ahead a stationary hand when judged at the moment of a short vibration (“haptic flash”) that is applied when the two hands are spatially aligned. We further investigated the haptic FLE. First, we compared participants’ performance in two isosensory visual or haptic conditions, in which moving object and flash were presented only in a single modality (visual: sphere and short color change, haptic: hand and vibration), and two bisensory conditions, in which the moving object was presented in both modalities (hand aligned with visible sphere), but the flash was presented only visually or only haptically. The experiment aimed to disentangle contributions of the flash’s and the objects’ modalities to the FLEs in haptics versus vision. We observed a FLE when the flash was visually displayed, both when the moving object was visual and visuo-haptic. Because the position of a visual flash, but not of an analogue haptic flash, is misjudged relative to a same visuo-haptic moving object, the difference between visual and haptic conditions can be fully attributed to characteristics of the flash. The second experiment confirmed that a haptic FLE can be observed depending on flash characteristics: the FLE increases with decreasing intensity of the flash (slightly modulated by flash duration), which had been previously observed for vision. These findings underline the high relevance of flash characteristics in different senses, and thus fit well with the temporal-sampling framework, where the flash triggers a high-level, supra-modal process of position judgement, the time point of which further depends on the processing time of the flash.

## Introduction

**Fig 1:**
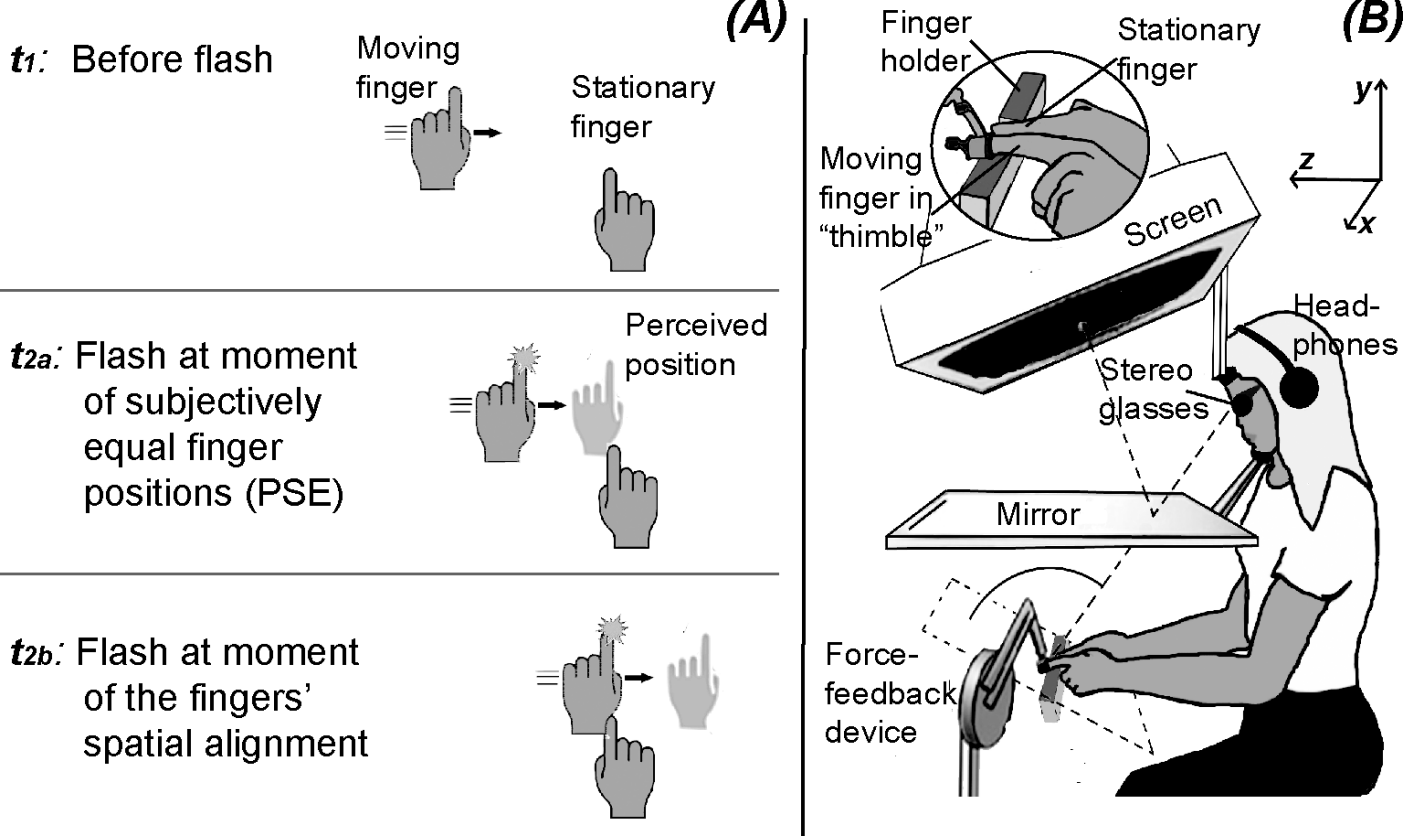
Haptic flash-lag effect and experimental setup. (A) A haptic flash-lag effect. One finger moves parallel to the transverse (x-) axis (t_1_). Then, the “haptic flash” is applied. The two fingers are perceived to be aligned during the flash, when the moving finger has not yet crossed the non-moving finger (t_2a_), but under spatial alignment the moving finger is perceived to be ahead the non-moving one (t_2b_). (B) Visuo-haptic set-up; the inset shows the finger holder that held the non-moving finger below the midpoint of the movement corridor. In the first experiment the moving finger was always the right index finger, in the second experiment both index fingers were used as moving finger in alternation.

In the flash-lag effect (FLE), a moving stimulus is seen to be ahead a brief visual stimulus that appears in alignment with the moving stimulus [1–3]. The FLE has been intensively studied, but rarely with regard to other modalities than vision (review in [4]). Some studies examined how additional haptic information and the participants’ control of the moving item or the flash appearance affect the *visual* FLE [5–8]. Occasionally, for visual-auditory and auditory stimulus conditions, effects were found that resemble the visual FLE [9–11] Recently, an only haptic flash-lag effect was observed (called buzz-lag effect in the study in [12]): Participants moved one hand in synchrony with a metronome back and forth parallel to the transverse body axis. The other hand was placed under the midpoint of the moving hand’s trajectory and did not move. A brief tactile stimulus was presented to the index finger of the moving hand at an unpredictable moment. The participants’ task was to decide whether at that time point the moving hand was ahead or behind the non-moving hand. The moving hand was *physically* behind the non-moving hand, when the two hands were *perceived* to be spatially aligned, indicating that the moving hand is perceived to be ahead the non-moving hand under physical alignment (Fig 1A). Potentially linked to this haptic flash-lag effect are mislocalizations of stimuli that are applied to the unseen hand during its movement [13–15]. In the visual FLE, both moving objects and non-moving objects are perceived to be shifted in direction of motion, but the mislocalization of the moving object is stronger [16,17].

A number of explanations have been suggested for the visual FLE: explanations based on differential latency [18, 19], on attention shifts [20], or on motion extrapolation [1]. Many of these explanations fail to account for auditory and haptic FLE effects [9, 10, 12], because they are based on particularities of visual processing [8, 18, 21–26]. Cellini and colleagues [12] argued that findings of FLEs in different senses and of cross-sensory effects indicate that such effects are likely due to supramodal or attentional processes rather than to sensory processes at a lower level. Corresponding accounts of the FLE include temporal integration [27], postdiction [28], or temporal sampling [29]. From their results, Cellini et al. [12] favored the temporal-sampling hypothesis, which suggests that the flash specifies when the position of the moving object is sampled [30]. The initiation of sampling requires time, and, hence, the position of the moving object is sampled only after the flash had appeared and it is shifted in direction of motion. The temporal-sampling hypothesis parsimoniously explains the FLE as a result of the processes directing attention towards the stimulus that initiates the position judgment, and thus predicts a high relevance for flash characteristics for the FLE.

In the present experiments, we investigated the relevance of flash characteristics for the haptic flash-lag effect. We assessed the haptic flash-lag effect using the methods by Cellini et al. [12]. In the first experiment we directly compared the haptic and the visual FLE under similar display conditions, and disentangled effects of the flash’s sensory modality and of the moving object’s modality. We assessed participants’ performance in four experimental conditions: a visual isosensory condition, where both the moving and the flashed object were presented only in the visual modality, a haptic isosensory condition where both objects were presented only in the haptic modality and two bisensory conditions. In the bisensory conditions the flash was presented only visually or only haptically, whereas the moving object was presented to both modalities simultaneously. Bisensory conditions were included to dissociate modality effects of the flash from modality effects of the moving object. At first glance, it may appear more straightforward to use instead cross-sensory conditions, in which the objects are in one modality and the flash is in another modality. However, this comes along with a dissociation of the positions of flash and moving objects in the cross-sensory, but not the isosensory condition. In pilot studies, this dissociation turned out to be highly confusing for the participants. Hence, we chose to focus our investigation on the comparison between iso- and bisensory conditions.

The isosensory visual condition was matched to the other conditions by displaying a small moving object on the screen that moved along a trajectory that corresponded to a previous finger movement of the participant. The non-moving finger was replaced by a non-moving visual object. Similar to the haptic flash, the visual flash was applied to the moving object, implemented as a color change. In [31] it had been shown that a considerable change in the moving object’s size can eliminate the visual FLE, if the participants perceive that two different objects are present before and after the change. However, the haptic flash in the present study did not disrupt the perception of a continuous identity of the moving object, and thus should not interfere with the FLE. We expected to observe both a haptic and a visual FLE with this design, and aimed to disentangle modality effects of the flash from modality effects of the moving object, by combining either a haptic or a visual flash with bisensory moving objects. In the second experiment, we tested the influence of flash intensity and flash duration on the haptic flash-lag effect. The visual FLE has been shown to increase with decreasing flash intensity and duration [18, 32], which we expected to observe for the haptic FLE as well.

## Experiment on haptics vs. vision

### Methods

#### Participants

We tested 8 participants (3 males, 5 females; age range: 20–27; mean age: 22). Participants were recruited in the year 2012 by an email send via a mailing list to all psychology students. The participants were from Giessen University, right-handed. Participants were only included when they had corrected-to-normal or normal visual acuity, unimpaired stereo vision and unimpaired motor and sensory functioning of their hands according to self-report. The number of 8 participants was chosen based on previous experiences with studying the haptic flash-lag effect [12]. Participants were naïve with regard to the aims of the study; they obtained course credit for their participation. Methods and procedures in all experiments reported were approved by the local ethics committee (LEK) of FB 06 at Giessen University and were in accordance with the ethical standard laid down in the Declaration of Helsinki (2008). Participants provided their written informed consent.

#### Apparatus and stimuli

We used a visuo-haptic setup (Fig 1B) that comprised an LCD screen (22”, 1024 x 1280 pixels, 120Hz, Samsung SyncMaster), a force-feedback device (resolution: 1000 Hz temporal, 0.03 mm spatial; workspace: 38 x 27 x 20 cm^3^; PHANToM 1.5A), stereo glasses (wireless; Nvidia), and headphones. Participants looked through the stereo glasses and mirror at the computer screen, (viewing distance, eye to mirror plus mirror to screen: 40 cm). Due to the mirror, participants were not able to see their hand and the mirror allowed to spatially align the haptic and the visual scenes, in particular the 3D positions of the fingers and the visual objects. Head movements were limited by a chin and a head rest. A holder that resembles a thimble connected the force feedback device to the index finger of the moving hand. The index finger of the non-moving hand was held stationary below the midpoint of the movement path by a “finger holder” (Fig 1B, inset). The distance between these devices and the participant’s body resulted in the participants’ arms being fully extended and not touching each other. The stimulus presentations, the sampling of responses, finger positions and forces and all the devices were controlled by a custom-made software on a PC.

Participants moved their index finger rhythmically back and forth parallel to the transverse axis (x in Fig 1B), or they watched corresponding transverse movements of a visual stimulus. A virtual force corridor (350 mm length, x-axis; 5 mm depth, z-axis) rendered by the PHANToM device limited the finger movement. The finger holder was 43 mm directly below the midpoint of the corridor. The PHANToM also produced a short vibration (33 ms duration, 100 Hz, sine-wave, nominally 5 N force amplitude) that was used as “haptic flash” and applied to the moving finger. This “haptic flash” acted in a direction perpendicular to the transverse movement path, parallel to the y-axis.

A red sphere of 5 mm diameter that was displayed on a black background (~0.7° visual angle) was used as visual moving stimulus. Its 3D-trajectory corresponded to a current or a previously recorded trajectory of the moving finger (~43° visual angle). Additionally, a stationary red sphere of 5 mm diameter was displayed at the 3D-position of the stationary finger’s holder in all conditions except for the isosensory haptic one. A visual flash was generated by changing the moving sphere from red to white for 33.3 ms (4 frames). Sparse further visual information guided the participants through the experiment. Via headphones we presented brief instructions and white noise (45 dB) to cover mechanical sounds from the PHANToM, as well as metronome signals (sine-wave, 698Hz, 20ms; 50dB) and movement feedback sounds (“ding” sound, Microsoft^TM^) when the finger of the participant reached the endpoint of the movement path.

#### Design

While participants produced or watched periodical movements, a flash was presented. Below the midpoint of the movement corridor was a non-moving object. The flash was presented on the moving object and thus separate from the non-moving object. The within-participant design comprised four experimental conditions: we varied the sensory modality of the flash (visual vs. haptic) and the sensory modalities of the moving or non-moving objects (isosensory vs. bisensory). In the isosensory conditions, both objects were presented in the modality of the flash (i.e., when the haptic flash was presented the objects were the two index fingers, and when the visual flash was presented the objects were visual spheres). In the bisensory conditions, the moving objects and non-moving objects were the two fingers combined with visual spheres that were spatially aligned with the fingers, and the flash was given in a single modality only. In the present experiment the right index finger was the moving finger.

The participants’ task was to judge whether the moving object was right or left of the non-moving object at the time point of the flash onset. The flash onset was at one out of seven positions along the movement path: -99, -66, -33, 0, 33, 66, 99 mm. Negative values indicate positions before the non-moving object had been crossed by the moving object. We combined the method of constant stimuli with a two alternatives forced choice paradigm (2AFC) and assessed the point where the non-moving and the moving object were perceived to be in spatial alignment (PSE = point of subjective equality).

#### Procedure

**Bisensory trials.** At the beginning of each trial, participants were reminded to position their left index finger in the finger holder and a straight line representing the target trajectory was visually displayed (length 290 mm corresponding to screen width). To initiate the movement, participants laid down the moving finger in a marked start area. Then, a female voice (recording) informed participants whether the movement would start at the right or the left side, and the visual scene went black. The start area was centered at the target trajectory and participants moved the moving finger as instructed by the voice to the extreme left or right side of the target trajectory. When the finger reached this position, metronome signals (each 667 ms; 1.5 Hz) and white noise were played. The visual objects (moving stimulus and stationary red sphere) appeared on the screen. Participants were allowed to start finger movement only after two metronome beats. They were instructed to move back and forth between the starting point and the target trajectory’s other side in synchrony with the metronome beats. Movement in one direction was considered a stroke, and when the finger approached a turning point (130 mm left or right from midpoint; i.e. 15 mm before turning point), feedback tones (“ding” sound, Microsoft^TM^) were provided.

During the 3rd or the 5th stroke a flash was presented: a visual flash in the bisensory-visual condition and a haptic flash in the bisensory-haptic condition. After completion of this stroke, sounds stopped and the visual scene went black, again. Participants had to judge by a specified finger movement (first to extreme left/right, then up) whether the moving object had been right or left to the non-moving one, when the flash had appeared. In case of a “movement error” (see below), a written message informed about the error, a trial was stopped before the participant’s response, and the trial was repeated later in the block.

**Isosensory-haptic trials.** A single trial in the isosensory-haptic condition was identical to a trial in the bisensory-haptic condition except for that no visual objects (moving stimulus and stationary red sphere) were presented.

**Isosensory-visual trials.** At the beginning of an isosensory-visual trial participants positioned their left index finger on their lap. The starting area for the right index finger was at a random x-position along the target movement path. In this condition, the right index finger was kept in the starting area by forces from the force-feedback device, and after 2 seconds a finger movement from a previous trial (see below) was redisplayed by a moving visual object. In addition, the stationary visual object and all auditory signals that accompany other types of trials were presented.

**Overall procedure of the experiment.** A single block of the experiment comprised two repetitions of each combination of flash position, starting point of the movement, and stroke at which the flash occurred for a single experimental condition. There were 3 blocks for each of the four experimental conditions, resulting in 4 [conditions] X 7 [flash position] X 2 [starting points] X 2 [flash stroke] X 2 [repetitions] X 3 [blocks] = 672 trials. Within each block, trials were presented in a random order; the three blocks of each experimental condition were presented successively with short breaks between the first and second blocks and between the second and third blocks, and the order of experimental conditions was balanced across participants according to a Latin square design. The experiment was conducted in two sessions each lasting about 2.5 hours.

At the beginning of each session of the experiment, participants trained the finger movements. In training trials, no visual information on the movement was provided and no flash was applied. When the participant had produced less than 17% movement errors in the past 20 trials the training phase was over. After the training phase in the first session, participants performed another 56 trials without visual feedback and flash. Finger trajectories recorded from these trials were later visually displayed in the isosensory-visual condition. The root-mean squared error (RMS) between measured and target parameters of movement defined the movement errors (6%). The RMS had to be below 45% both for stroke length (target: 290 mm) and duration (target: 667 ms).

#### Analysis

First, the single responses of the participants were coded as “behind” or “ahead” judgments on the moving object compared to the non-moving object. We calculated for each condition the individual percentage of trials in which the judgment was “ahead” as a function of the position of the moving object at flash onset. Using the psignifit toolbox [33], we fitted cumulative Gaussian functions to these data, and assessed for each participant and experimental condition the PSE (point of subjective equality) from the function’s parameter μ and the JND (just noticable difference) from σ (cf [34, 35]). The PSE corresponds to the moving object’s position at flash onset that is randomly judged to be ahead or behind the non-moving object. Thus, it corresponds to the moving object’s position that is subjectively equal to the position of the non-moving object. A PSE significantly less than 0 mm indicates a flash-lag effect, because it indicates that the moving object is physically behind the non-moving object. The JND, when assessed from σ, is the difference between the PSE and the moving object’s position at flash onset when it was judged to be ahead the non-moving object 84% of the time [33]. JNDs assess judgment precision.

The experiment is designed to assess the FLE based on position. However, the FLE temporal extent has regularly been reported in previous studies and is of high relevance for a number of theories of the FLE (e.g., [18, 19, 27]). Therefore, we further calculated for each trial the difference between flash onset time and the time when moving object and non-moving object were physically aligned (“flash-onset time”: values were negative when at flash onset the moving object was physically behind the non-moving one). We recalculated PSEs based on response data that were binned according to flash onset times (bin width 20 ms).

Data were analyzed using a limited number of hypothesis-driven planned comparisons, which were−as it is common practice−not adjusted for the number of tests (e.g., [36], for a different view on this topic see [37]).

### Results

#### PSEs computed from flash position and time

**Fig 2:**
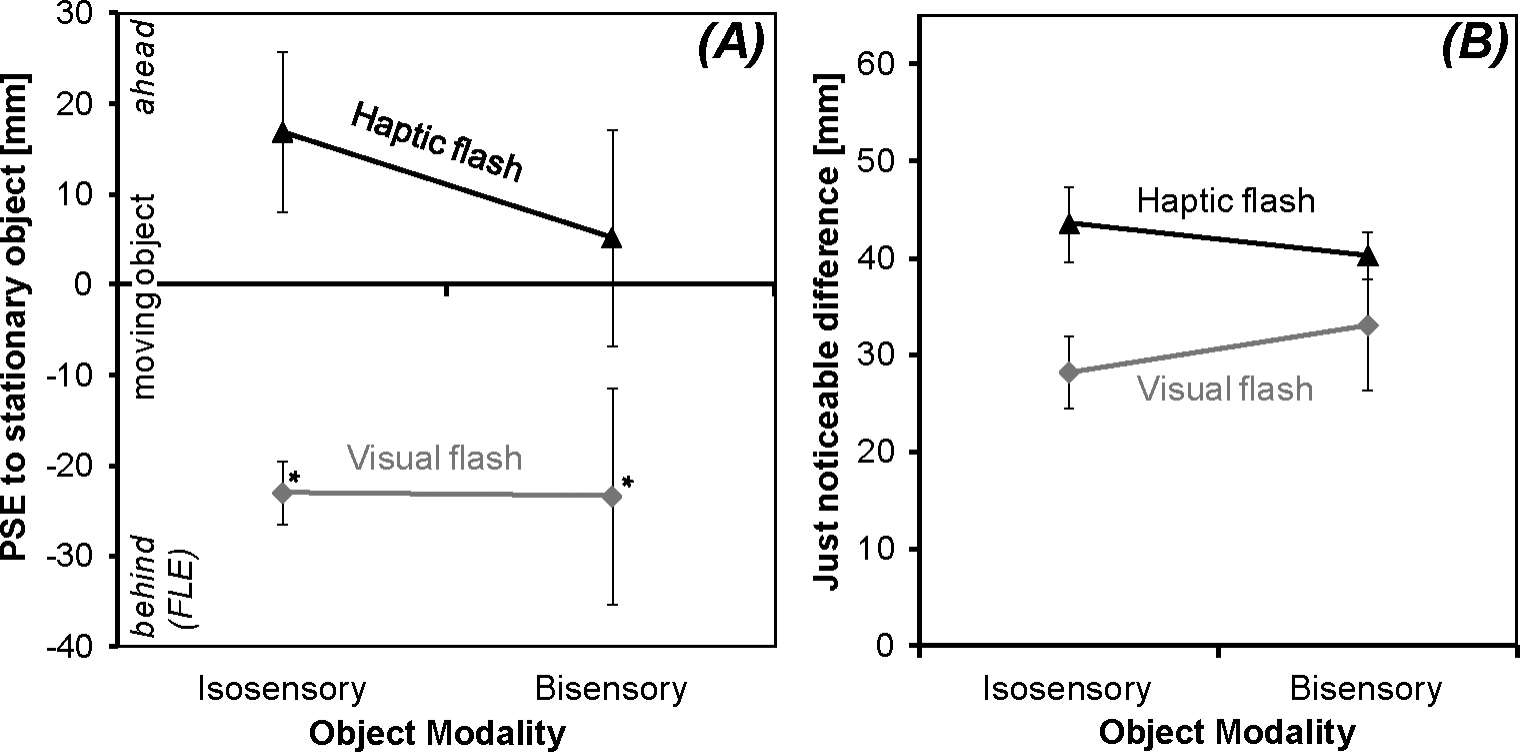
Results, first experiment. (A) Average point of subjectively equal position of the moving object to the non-moving, stationary object (PSE) and (B) judgment precision as assessed by JNDs computed from flash onset position for visual and haptic flashes combined with isosensory (visual or haptic) and bisensory (visuo-haptic) objects. A significant negative PSE (marked by an asterisk) indicates a flash-lag effect. Error bars represents standard errors of the means.

Individual PSEs were entered into ANOVAs with the within-participant factors Flash Modality (haptic vs. visual) and Object Modality (isosensory vs. bisensory). The PSEs calculated from flash position (Fig 2A) were smaller for the visual than for the haptic flash (-23 vs. 11 mm), *F*_*1*,*7*_ = 13.66, *p* = .008, *ɳ*_*p*_^*2*^ = .66. The objects’ modality had no significant effect (main effect: *F*_*1*,*7*_ = 0.98, *p* = .356, *ɳ*_*p*_^*2*^ = .12, interaction: *F*_*1*,*7*_ = 0.45, *p* = .522, *ɳ*_*p*_^*2*^ = .06). We further checked whether PSEs in the four different conditions were smaller than 0, which would indicate a FLE. This was observed as expected, when a visual flash was presented (isosensory: *t*_*7*_ = 6.60, *p*< .001; bisensory: *t*_*7*_ = 1.91, *p* = .049, one-tailed tests). In contrast, with a haptic flash PSEs were yet numerically larger than 0, which is not in line with a FLE. In two-tailed tests, haptic PSEs did not significantly differ from 0 (isosensory: *t*_*7*_ = -0.59, *p* = .57; bisensory: *t*_*7*_ = -1.42, *p* = .20). A sensitivity analyses conducted with G*Power 3 [38] revealed that a single-sided trend (α < .10) for a flash-lag effect would have been detected in this experiment with a power of more than 90% if the effect was 27 mm or higher (the standard deviation for these analyses was assessed from the average of the between-participant variance across the four experimental conditions as 28 mm). Similarly, a haptic FLE of similar magnitude as previously observed in [12], i.e. between 25 and 51 mm, should have been detected with a power between 88 and 99%.

As should be the case, PSEs from flash onset time showed the same pattern of results as observed for the PSEs from position: They were smaller with a visual flash (average: -34±9 ms, only isosensory: -36±5 ms, only bisensory: -33±18 ms; average ± standard error) than with a haptic flash (average: 17±14 ms, only isosensory: 9±13 ms, only bisensory: 24±18 ms), *F*_*1*,*7*_ = 15.27, *p* = .006, *ɳ*_*p*_^*2*^ = .69. Other effects in the ANOVA were not significant (Object Modality: *F*_*1*,*7*_ = 0.99, *p* = .353, *ɳ*_*p*_^*2*^ = .12, interaction: *F*_*1*,*7*_ = 0.23, *p* = .645, *ɳ*_*p*_^*2*^ = .03).

#### JNDs computed from flash position

Individual JNDs (Fig 2B) were also submitted to an ANOVA. JNDs were smaller with the visual than with the haptic flash (31 vs. 42 mm; main effect Flash Modality: *F*_*1*,*7*_ = 6.81, *p* = .035, *ɳ*_*p*_^*2*^ = .49), indicating more precise judgments with the visual flash. Other effects were not significant (main effect Object Modality: *F*_*1*,*7*_ = 0.05, *p* = .826, *ɳ*_*p*_^*2*^ = .01, interaction: *F*_*1*,*7*_ = 0.74, *p* = .418, *ɳ*_*p*_^*2*^ = .10).

### Discussion haptics vs. vision

We adapted the methods previously used to examine a flash-lag effect in the haptic sense to examine also the visual FLE [12]. These methods differ in several aspects from standard displays used to investigate the visual FLE (motion had ac- and deceleration profile rather than constant velocity, permanent non-moving object, flash applied to moving rather than to non-moving object). However, consistent with previous observations of visual FLEs when biological motion was displayed (e.g., [5, 39]) or when changes of the moving or stationary stimuli were used as a flash (e.g., [40–42]), we observed a visual FLE of typical magnitude (about 36 ms). However, we failed to replicate the haptic flash-lag effect itself: Moving finger and non-moving finger were perceived to be aligned approximately at the moment when they were physically aligned. That is, in the visual-only condition we observed a significant FLE, but not in the haptic-only condition. Most importantly, from the experimental findings, we were able to attribute the difference between the visual and the haptic condition entirely to the sensory modality of the flash, not to that of the moving or non-moving objects: Also with bisensory objects (finger plus visual representation) the visual flash but not the haptic flash induced a FLE, and the magnitude of the FLE did not reliably differ between isosensory and bisensory objects. We conclude that the haptic and the visual effects differ due to differences in characteristics of the flash, not of the objects. Results on judgment precision (assessed by JNDs) fit this view, in that also precision significantly only varied with the sensory modality of the flash (visual > haptic). These findings strongly emphasize the relevance of the flash’s characteristics. This does not directly support the notion that supramodal flash-triggered processes account for the FLE, but it is highly consistent with this view: If the flash is assumed to trigger the sampling of positions of the moving object, and the FLE is explained by delay in the initiation of sampling [30], the flash characteristics should be highly relevant for the initiation time point of sampling and thus the magnitude of the FLE.

But why did we fail to replicate the haptic flash-lag effect? A few methodological changes from the previous [12] to the present study might be responsible: a) The predictability of the flash was increased, in that we used one instead of two metronome frequencies and only the right finger moved instead of both fingers in alternation. Predictability of the flash can decrease the visual FLE [43]. However, the level of predictability in the present experiment did not eliminate the visual FLE, which argues against the hypothesis that high predictability (alone) eliminated the haptic FLE. b) The haptic flash was longer (33 vs. 20 ms), more intense (nominal 5 N vs 3 N) and of lower vibration frequency (100 Hz vs. 200 Hz), which for technical reasons (time required by device to build up the commanded force) most likely further increased differences in actual force transmitted to the finger. The visual FLE had been shown to decrease and even vanish with increasing flash intensity and flash duration [18, 32]. The second experiment, investigated the influence of flash intensity and duration on the presence of a haptic FLE. Predictability of the haptic flash was decreased as compared to the first experiment.

## Experiment on haptic flash duration and intensity

### Methods

#### Participants

11 healthy and naïve students from Giessen University participated for course credit; none of the participants had taken part in the first experiment. They were recruited in the year 2013 again via a mailing list. Inclusion and exclusion criteria were the same as in Exp. 1. Additionally, data from 2 participants had to be removed from analysis due to extremely outlying JNDs in some conditions (values deviated > 2.5 standard deviations from mean). The final sample included 8 females and 1 male with a mean age of 24 years.

#### Apparatus, stimuli, procedure, design, and analyses

We used the same apparatus as in the first experiment. The procedures in each single trial and the stimuli corresponded to those used in the isosensory-haptic conditions in the first experiment. So, during the entire experiment we did not provide any visual information about the moving finger’s position. In contrast to the first experiment, we used four different haptic flashes that were defined by sine-wave vibrations with durations of 17 and 33 ms, and nominal force amplitudes of 1.5 N or 3 N. The frequency was 60 Hz.

The design comprised two within-participants factors, Flash Intensity (weak vs. strong) and Flash Duration (17 vs 33 ms). The flash onset was at the positions -99, -66, -33, 0, 33, 66, 99 mm along the trajectory of the moving finger and the movement started on the left or the right side. In contrast to Experiment 1, the flash was given during the 2^nd^, 3^rd^, 4^th^ or 5^th^ stroke; in half of the trials, participants moved the left finger and the right finger remained stationary, whereas in the other half of the trials, participants moved the right finger and the left finger remained stationary. These variations were introduced in order to decrease the predictability of the flash. Each combination of the 4 experimental conditions, 7 flash positions, 2 movement start points, 4 flash times and 2 moving fingers was presented once in a session, yielding 4 x 7 x 2 x 4 x 2 = 448 trials per session. The left and the right finger alternatingly served as moving finger; the moving finger switched after every 56 trials. Whether a participant started the experiment with the left or the right finger being the moving finger was counterbalanced across participants. In all other aspects, trials were presented in random order. There were two sessions on different days, each lasting 3.5 hours. At the beginning of the first session were two training phases, one for the movement of the right finger and one for the movement of the left finger (see first experiment). Movement errors (5% of trials) were defined and data were analyzed as in the first experiment.

### Results

#### PSEs computed from flash position and time

**Fig 3:**
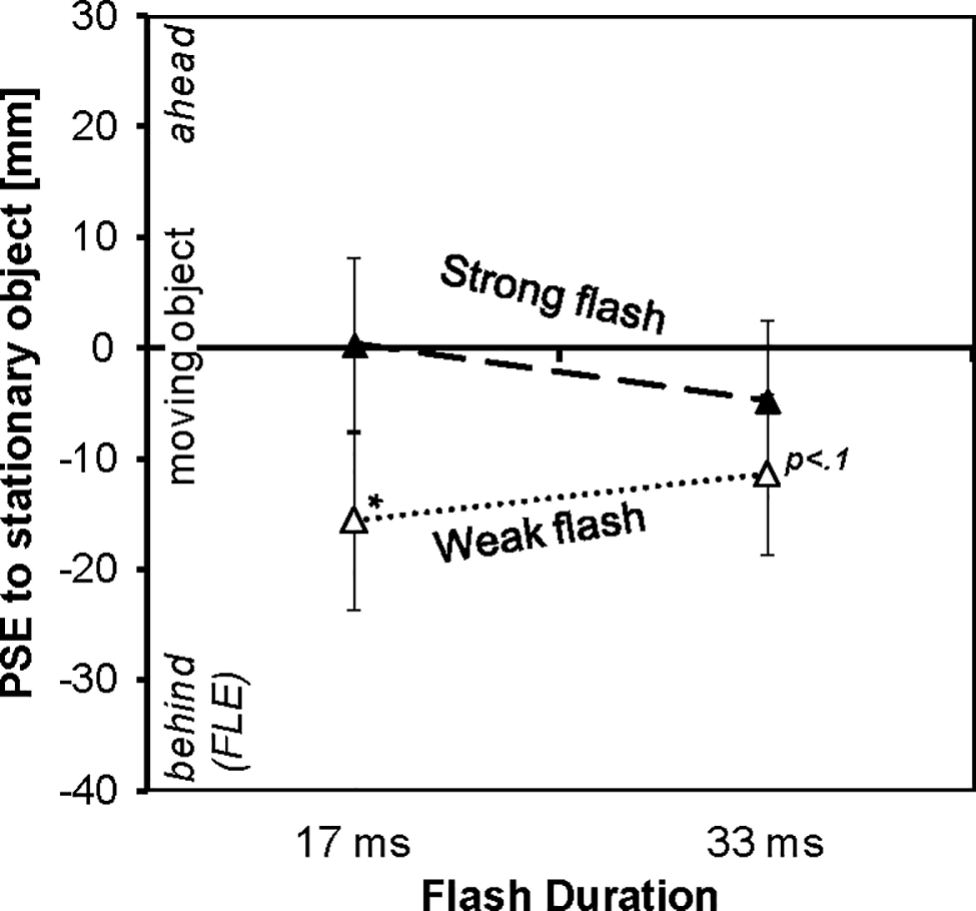
Results, second experiment. Average point of subjectively equal position of the moving to the non-moving, stationary object (PSE) computed from flash onset position as a function of the intensity and duration of a haptic flash. A significant negative PSE (asterisk) indicates a flash-lag effect. Error bars represents standard errors of the means.

Individual PSE were entered into ANOVAs with the within-participant factors Flash Intensity and Flash Duration. PSEs calculated from flash position (Fig 3) were smaller for the weak as compared to the strong haptic flash, *F*_*1*,*8*_ = 33.00, *p* < .001, *ɳ*_*p*_^*2*^ = .81. This intensity effect was more pronounced for the 17-ms flashes than for 33-ms flashes, interaction Flash Intensity X Flash Duration: *F*_*1*,*8*_ = 7.19, *p* < .028, *ɳ*_*p*_^*2*^ = .47. There was no main effect of Flash Duration, *F*_*1*,*8*_ = 0.03, *p* < .868, *ɳ*_*p*_^*2*^ = .00. In order to understand the interaction, we tested for the predicted effect of Flash Duration separated by Flash Intensity (one-tailed *t*-tests): For weak flashes the test was significant, *t*_*8*_ = 1.88, *p* = .048, which indicates that PSEs are smaller with weak 17-ms flashes than with weak 33-ms flashes; for strong flashes no effect of flash intensity was found, *t*_*8*_ = 1.35, *p* = .107. Condition-wise single-sided *t*-tests against 0 indicated a significant flash-lag effect for the weak short flash, *t*_*8*_ = 1.92, *p* = 0.046, a trend for the weak long flash, *t*_*8*_ = 1.57, *p* = 0.078, and no effect nor trend for the strong long flash, *t*_*8*_ = 0.65, *p* = .267. The PSE for the strong short flash was numerically larger than 0, which is not in line with an FLE; in a two-tailed test the value did not differ significantly from 0, *t*_*8*_ = 0.039, *p =* .969. A sensitivity analyses [38] revealed that a single-sided trend (α < .10) for a flash-lag effect would have been detected in this experiment with a power of more than 90% if the effect was 21 mm or higher (standard deviation assessed as 23 mm; 72% power for observed effect of 15 mm).

PSEs from flash onset time showed the same pattern of results as position PSEs (averages ± standard error for 1.5N-17ms: -16±12 ms; 3.0N-17ms: 5±11 ms; 1.5N-33ms: -10±10 ms; 3.0N-33ms: -2±11 ms; main effect Flash Intensity: *F*_*1*,*8*_ = 34.25, *p* < .001, *ɳ*_*p*_^*2*^ = .81; main effect Flash Duration: *F*_*1*,*8*_ = 0.01, *p* = .937, *ɳ*_*p*_^*2*^ = .00; interaction: *F*_*1*,*8*_ = 6.69, *p* = .032, *ɳ*_*p*_^*2*^ = .46).

#### JNDs computed from flash position

An ANOVA on the individual JNDs did not reveal any significant effect (Flash Intensity: *F*_*1*,*8*_ = 2.49, *p* = .154, *ɳ*_*p*_^*2*^ = .24; Flash Duration: *F*_*1*,*8*_ = 0.89, *p* = .374, *ɳ*_*p*_^*2*^ = .10, interaction: *F*_*1*,*8*_ = 4.94, *p* = .057, *ɳ*_*p*_^*2*^ = .38). The average JND was 50±2 mm (condition-wise average ± standard error for 1.5N-17ms: 58±4 mm; 3.0N-17ms: 45±3 mm; 1.5N-33ms: 48±3 mm; 3.0N-33ms: 50±4 mm).

### Discussion haptic flash duration and intensity

We varied the duration and intensity of the haptic flash. With a weak flash we observed a haptic flash-lag effect, with a strong flash the effect was not significant. Flash duration slightly, but significantly modified the influence of flash intensity. The haptic FLE observed with a weak flash was significantly larger when its duration is short as compared to long, whereas flash duration did not change the effects of a strong flash. The influences of haptic flash intensity and flash duration parallel findings from vision, that have shown that the visual FLE decreases with flash intensity and with the length of the trajectory of a moving flash (or flash duration), at least up to a flash duration of 80 ms [18, 27, 32, 44–46]. Thus, similar factors, specific flash characteristics, contribute to the visual and the haptic FLEs.

As compared to the first experiment, we decreased the flash intensity and the flash predictability. With a weak flash we observed a haptic flash-lag effect as did Cellini et al. [12], with a strong flash the effect was not significant, as in the first experiment. Thus, flash intensity can explain the differences between observations in the present Experiment 1 and in the previous study [12]. From the present data we cannot entirely exclude that predictability plays some further role for these differences. However, another observation strengthens the view that specific characteristics of the flash, such as duration and intensity, are of particular high relevance: The flash-lag effect with a weak short flash here was still considerably smaller than in the previous study (17 ms vs. 56 ms). The flash here and the flash in [12] mainly differ in nominal force (1.5 N vs. 3N) and in vibration frequency (60 Hz vs. 200Hz), while predictability was similar. For technical reasons the proportion of the nominal force transmitted to the finger with a frequency of 200 Hz is smaller than at 60 Hz, and thus the actual force at the finger was probably weaker in [12] than with the weak flash here, indicating again that differences in flash intensity parallel differences in FLE.

## General discussion

We observed a visual FLE under conditions in which the existence of a haptic flash-lag effect had been shown [12]: participants judged a moving object’s position compared to a non-moving object when a flash appeared on the moving object. The moving finger is consistently judged to be ahead, when the two objects are physically aligned. This is consistent with previous studies that found that actually both the non-moving and the moving object are judged to be ahead, as in those studies and the experiments reported here the moving object is judged to be ahead of the non-moving object [16, 17]. In the first experiment, the visual FLE was larger than the haptic one. We were able to attribute this difference to the sensory characteristics of the flash, because the effects did not vary with the sensory modality of the objects (haptic, visual, bisensory). The second experiment demonstrated that the haptic flash-lag effect increases when the haptic flash is less intense, and for a weak flash intensity the haptic flash-lag effect tended to be slightly smaller with a short as compared to a long flash. This parallels findings from the visual FLE regarding flash intensity and duration [18, 27, 32]. Taken together, our findings emphasize the high relevance of specific characteristics of the flash, but not of the characteristics of the moving object (nor of the non-moving one, relative to which the position judgement was given). These outcomes fit with the idea that a supramodal flash-triggered process, which follows similar rules independent of modality, can account for the FLE.

Flash characteristics that influenced the FLE in the present study can be linked with processing times for the flash: From reaction times, it is known that processing is faster for stimuli (including visual and haptic stimuli) that are more intense and that are of longer duration—the latter in particular if the stimuli are of low intensity and the durations do not exceed the range of tens of milliseconds [47]. Furthermore, increasing the stimulus intensity or duration enhances its perceptual salience, so that more salient or better visible objects are processed faster than less salient ones (e.g. in Hess effect [48]). Indeed, the visibility of a stimulus can affect relative position judgements and the magnitude of the FLE [32, 49]. Accordingly, strong flashes in the second experiment might have been processed faster than weak ones, the weak long flash might have been processed faster than the weak short flash, but flash duration should have hardly affected processing time for the strong flash. A flash that is processed faster will trigger the localization of the moving stimulus at an earlier point in time and thus closer to its position at flash onset. This reduces the FLE—in line with the observed pattern of results. A similar argument can explain why the visual FLE in the first experiment was larger than the haptic FLE, given that haptic stimuli—at least by and large—tend to be processed faster than visual stimuli [50]. Taken together, our results support the view that the flash triggers a high-level supra-modal process of position judgement, the time point of which further depends on the processing time of the flash (cf [4, 30]).

The results can be taken as evidence against several specific theoretical accounts of the FLE. As already detailed in the Introduction, they argue against any account that associates the FLE with visual processes. Furthermore, results may not be consistent with the differential latency theory [19, 46]: This theory assumes that processing time is shorter for a moving than for a non-moving stimulus, i.e. the flash in the standard display. Hence, when observers become aware of the flash, the percept of the moving stimulus refers to a time point after the physical onset of the flash, shifted in movement direction. In our experimental design, the flash was displayed on the moving object (implemented as a brief vibration on the moving finger or as color change of the moving sphere), therefore differential latencies for moving and stationary stimuli do not seem a reasonable account of the observed effect. Moreover, the theory predicts that processing times of both the flash and the moving object influence the FLE. Here we observed a consistent influence of the manipulated flash characteristics (modality, duration, intensity), but we did not observe an influence of object characteristics that are well-known to affect processing time: Several studies show that bisensory stimuli are processed faster than unisensory ones [51, 52], but the FLE did not differ between iso- and bisensory objects.

Along the same lines of reasoning, our data are also inconsistent with the motion extrapolation hypothesis, which assumes that the perceived trajectory of a moving object is extrapolated forward to compensate for neural delays and favour efficient interceptive behaviour [1, 6, 26, 53]. Nijhawan and Kirschfeld [6] proposed that motor forward models and visual extrapolation are analogous mechanisms that compensate for specific neural delays and subserve the purpose of successful motor behaviour. Within this framework, one should expect a difference between bisensory and isosensory conditions, or at least a precision increase in the bisensory conditions, as variance in the final estimate should be minimized when visual and haptic cues are combined, as it is regularly observed in similar situations of multisensory integration [34]. We clearly did not observe such an outcome.

Our results are, in addition, at odds with the idea that an attention shift from the moving object towards the flash underlies the FLE, because the flash was given on the moving object and spatial attention needed not to be shifted [12, 20]. A similar argument against a role for attentional deployment has been previously made from observations of visual FLEs when interleaving or spatially aligning flash and moving objects [42, 54]. Finally, the results may not be consistent with the postdiction theory [28, 55]. According to this theory localization is based on integration of stimulus positions over a period of time, and the flash can “reset” this position integration; the extent of reset increases with the salience of the flash. As a consequence of the reset, localization of the moving stimulus refers to positions after the flash had appeared, and the moving stimulus is perceived to be shifted in movement direction. With more salient flashes, the FLE should increase. In contrast, we observed that the FLE decreased with a stronger, and probably more salient, haptic flash. However, the predecessor of postdiction theory, namely temporal integration [27] might be able to account for our findings: Similar to temporal sampling, [29] it explains the FLE by assuming that the flash starts a temporal window of about 600ms in which position information about the moving stimulus is collected. However, the implausibly long window of temporal integration has cast doubts on this approach [56].

Few other studies have investigated non-visual or crossmodal FLEs: Complimentary to our finding of a FLE with a visual flash and visuo-haptic objects, in [6] a cross-modal FLE with a visual flash and a haptic object has been reported. In that task, participants wielded an unseen rod with their hand and judged its position with respect to a visual flash. In [11] it has been shown that the visual FLE is reduced and judgment precision increased by a bisensory flash, namely when a brief tone accompanies the flash. When a tone was presented briefly before the flash the FLE further decreased, whereas the FLE increased with a tone briefly after the flash. The results have been interpreted in terms of an attraction of the time point of awareness of the flash towards the tone. A later tone delays the time point of flash awareness, increasing the FLE and vice versa for an early tone. This finding fits our view that the flash induces a supra-modal relative position judgment, the starting time of which depends on flash awareness.

In a visual FLE experiment in [57] the flash was replaced by an auditory click that marked the time point when the position of the moving visual object had to be compared to a stationary fixation cross. In this cross-sensory condition, the researchers observed a flash-lead rather than a flash-lag effect, indicating again the relevance of characteristics of the flash. Finally, in [9] a moving auditory object was combined with a stationary auditory flash (a beep). The authors observed a large auditory FLE (~200 ms) that clearly exceeded a visual FLE (~25 ms) observed in another condition. In addition, the researchers combined moving auditory objects with stationary visual flashes and moving visual objects with stationary auditory flashes. The FLE in the cross-sensory conditions was in-between the FLEs in the unisensory conditions. Results from [9] on the size of the audio-visual FLE are at odds with the audio-visual flash-lead effect observed in [57]. In addition, the differences between cross-modal and uni-modal conditions with the same type of flash in [9] may imply an influence of the objects’ sensory modality on the FLE that we did not observe. However, the task in [9] differed from that in the present study and in [57]: Here and in [57] moving objects and non-moving objects were always presented in the same sensory modalities, and thus the comparison of their positions was never a purely cross-sensory task. Only the sensory modality of the temporal marker, the flash, could differ. In contrast, cross-sensory conditions in [9] implied a cross-sensory comparison of positions, and unisensory conditions a unisensory comparison. Probably, it is the difference between within-sensory and cross-sensory position tasks rather than the objects sensory modality that explains differences between unisensory and cross-sensory conditions in [9], and thus the seeming contrast to the present results. However, these are speculations that remain to be tested.

Overall, we replicated the haptic flash-lag effect observed in [12] and demonstrated a visual FLE under equalized display conditions. In particular, the characteristics of the flash (sensory modality, intensity, duration) determined the magnitude of the FLE. The results corroborate the view that the flash triggers a high-level supra-modal judgment of the moving object position relative to the flash, the time point of which further depends on the processing time of the flash [4, 30]. By preclusion of other alternatives, temporal sampling might be a good candidate for such a supra-modal process [29]. Other findings on nonvisual and cross-modal flash-lag effects are mainly consistent with this view. However, future studies are required to further investigate how similar flash-lag effects in different senses really are.
